# The Forms and Structures of Chimpanzee Algae Fishing

**DOI:** 10.1002/ajp.70164

**Published:** 2026-06-08

**Authors:** Charlotte Wiltshire, Erin G. Wessling, Liran Samuni, Catherine Hobaiter

**Affiliations:** ^1^ Wild Minds Lab, School of Psychology & Neuroscience University of St Andrews St Andrews UK; ^2^ Cooperative Evolution Lab Deutsches PrimatenZentrum GmbH Goettingen Germany; ^3^ Cooperative Cultures Research Group Max Planck Institute for Evolutionary Anthropology Leipzig Germany; ^4^ Department of Human Origins Max Planck Institute for Evolutionary Anthropology Leipzig Germany

**Keywords:** action grammar, chimpanzee, *Pan troglodytes verus*, repertoire, sequences, tool use

## Abstract

Variation in the expression of behavior is a critical measure for understanding how socio‐ecological factors shape cognitive and behavioral evolution and adaptability. Detailed descriptions of behavioral repertoires and how they are combined and structured into programs of actions is an essential foundation for this work. However, comparisons within and across species are made challenging where there is substantial variation in the level of detail at which behaviors are described. Here, we use a systematic, multi‐level framework to describe a recently reported chimpanzee tool use behavior—algae fishing—at three levels of granularity: Functional Behavioral Categories, Behaviors, and Behavioral Elements. We then describe how these units are combined into structured programs of action. Despite variation in the detail at which tool use behaviors are described in the literature, we suggest that chimpanzees' algae fishing repertoire is relatively large, as compared to other forms of chimpanzee tool using, and flexibly deployed at each level of description. The varied use of techniques by adults suggests that there is no single optimal solution for algae fishing, and that chimpanzees benefit from maintaining multiple strategies for this dynamic foraging challenge. We provide an example of a structured framework that can be applied to describe different levels of detail and used to show within‐ and between‐task variation. Systematic frameworks that can be consistently applied across species and contexts are critical for providing the like‐with‐like comparisons necessary for robust investigations of species‐level cognition and behavior.

## Graphical abstract

We use a systematic, multi‐level framework to describe a recently reported chimpanzee tool use behavior—deep‐water algae fishing. Moyen‐Bafing chimpanzees combined 5 Grips and 4 Actions into 22 Techniques. Our data suggest that chimpanzees benefit from maintaining multiple flexible strategies for this dynamic foraging challenge.

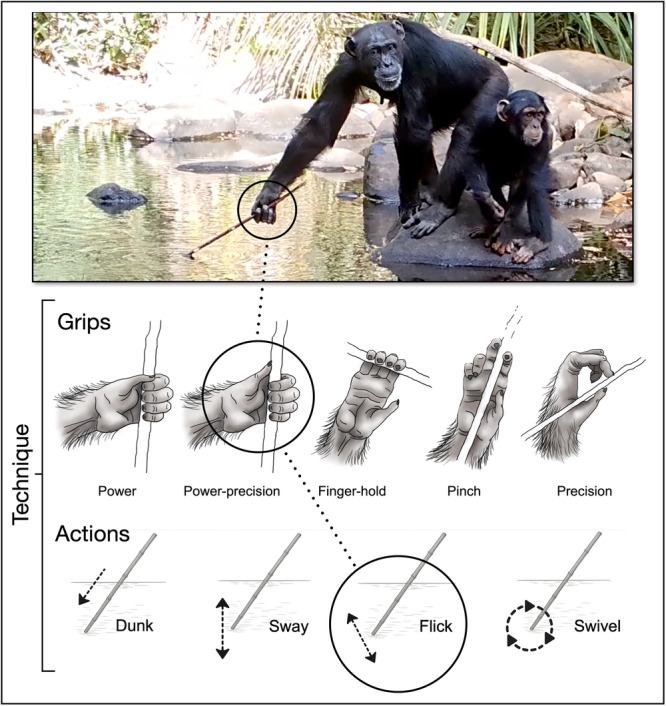

## 1 Introduction

Behavioral flexibility enables individuals to adapt to the demands of both their physical and social worlds, allowing them to survive (and sometimes thrive) in dynamic and diverse environments. However, not all behaviors are variably expressed. Just as selection can act on the evolution of highly complex structures (such as the eye; Darwin 1859), it can lead to the production of highly complex, but fixed, patterns of behavior—for example, intricately executed mating performances (Tinbergen 1951; Griffith and Ejima 2009; Fusani et al. 2014; Scholes et al. 2017; Wessling et al. 2026). Variation in behavior can be shaped directly by features of the local environment—for example, material availability (van Berkel et al. 2024; O'Malley et al. 2025). But, in some cases, behavioral variation reflects decisions made by an individual that integrate information across multiple features of a problem. These can include tailoring tools to problems (e.g., retrieving one tool to obtain another that accesses a reward; Wimpenny et al. 2009), adjusting tool length to reward depth (Hunt et al. 2006), matching tool characteristics (e.g., brush tips) to the insect species targeted (Sanz et al. 2014), expressing individual kinematic preferences or abilities (Estienne et al. 2017; Poncet et al. 2022), selecting materials based on their structural flexibility (Bailey et al. 2014), or adjusting construction materials and methods according to nest type (Permana et al. 2024). The ability to integrate information and choose flexibly from among multiple viable solutions to a problem (variants, cf., Wessling et al. 2026) is associated with heightened cognitive demands; for example, increasing load on working memory and the recruitment of executive functions, such as mental set‐shifting and inhibition (Uddin 2021; Haidle 2010; Cantwell et al. 2022).

The granularity (level of detail) with which we describe behavior influences our ability to detect (or overlook) potential variation in the expression and use of behaviors and our understanding of the ways in which behaviors are combined and ordered (Byrne and Byrne 1993; Mielke et al. 2024). Variation in the degree of detail at which a behavior is described may result in substantial differences in the variation recorded by the observer both within and between individuals—so much so that studies of the same task, coded to different degrees of detail have been used to argue both for (Byrne and Russon 1998; Byrne et al. 2011) and against (Tennie et al. 2008) capacities such as social imitation in nonhuman species. At the same time, not all forms of potentially perceptible variation are relevant to every research question (e.g., gripping a tool between the second and third, or second and fourth digits might be functionally equivalent, despite differing in form), and very detailed cataloging of all forms of every detectable variation of a behavior represents a substantial time‐burden (Grund et al. 2024). Nevertheless, there is likely some level of detail and systematic structure that warrants consistent application across studies, particularly when a behavior is relatively new, and our understanding of which features are relevant remains limited. Systematic frameworks of behavioral description allow more robust understanding of relative repertoire sizes, repertoire overlap (both within and across species), and cognitive abilities, which in turn enable systematic, widespread comparison of behavioral units both across and within tasks and species (Wessling et al. 2026).

Tool use is a taxonomically widespread example of a behavior that often (though not always) requires behavioral flexibility for successful execution. Individuals can express behavioral flexibility by varying their use of elements from within a set (or “repertoire”) of options—the larger the set, the greater the opportunity for behavioral flexibility, but often also the greater the cognitive challenge (Sambrook and Whiten 1997; Coolidge and Wynn 2001). In humans, the selection and combination of behaviors for a given task depends on factors such as the specific physical components of the problem, the individual's level of expertise, and personal or cultural preferences (Keen 2011; MacDonald et al. 2017). But, in addition to choosing from within a repertoire of units, many behaviors also require multiple steps that need to be executed in a particular order to be successful (Parker and Gibson 1977; Sellet 1993; Byrne et al. 2001; Byrne 2002; Fragaszy et al. 2023; Wimpenny et al. 2009). As a result, where there are sequences of steps, another way in which individuals may respond to a task with behavioral flexibility is by varying the order and structure in which they complete these steps. These steps can be described together as a behavioral *program* of actions (Byrne and Russon 1998; Haidle 2010). If the *repertoire* of units is like an ingredients list of action items, the *program* is the recipe, i.e. how they are combined (Byrne and Russon 1998). The collective structure of the program, such as the ways in which the program is ordered and/or how steps (or sections of steps) are repeated, etc., is its *action‐grammar* (Payne and Green 1986; Pastra and Aloimonos 2012; Hayashi 2015).

The human ability to stack components into recursive, hierarchically‐structured sequences is most richly expressed as syntax (or grammar) in language (Fitch et al. 2010; Hurford 2011; Lashley 1951; Pastra and Aloimonos 2012; Stout and Chaminade 2012) and music (Fitch 2013; Fitch and Martins 2014). But a similar ability to order and iterate steps can be seen across many tasks (or programs of action), from boiling a kettle to driving a car (e.g., Ambrose 2001). Non‐human animals are also capable of hierarchical organization, or action‐grammars, in their behavioral programs (Byrne and Russon 1998; Bergman et al. 2003; Suzuki et al. 2006; Carvalho et al. 2008; Haidle 2010; Sainburg et al. 2019; Mielke and Carvalho 2022; Howard‐Spink et al. 2024; Lameira et al. 2024; Wimpenny et al. 2009). Where this order can be varied flexibly case‐by‐case, with the ability to execute later steps depending on earlier choices, these programs have been suggested to indicate mental organization and the ability to plan ahead (e.g., to retrieve the targeted reward at Step 3, one must first not only complete Steps 1 and 2, but do so in such a way that already anticipates the need for Step 3; Russon 1998; Stokes and Byrne 2001; Sanz and Morgan 2013).

Typically, the greater the number of “steps” in the *program*, or the longer the delay before achieving the goal (or reward), the more tenuous the connection between earlier steps and their eventual outcome, increasing the cognitive challenge of planning how to obtain it. For example, the acquisition of a tool (often required towards the beginning of a tool behavioral *program*) could occur once an individual has already arrived at a food resource that requires a tool, but could also be anticipated or even planned for prior to arrival, i.e. where an individual collects a tool on the way to the food source (e.g., Boesch and Boesch 1984; Almeida‐Warren et al. 2017). Arriving to a tool use location with a tool suggests that the individual has a mental representation of their future‐orientated behavior, including both the target and the tool required to access it (Byrne et al. 2013; Osvath and Martin‐Ordas 2014; Musgrave et al. 2024). The rules by which programs can be completed (the action grammar)—for example the number of steps, whether steps are optional, and whether steps or sections can be repeated—offer insight into the cognitive challenges faced to reach a desired outcome.

As with describing behavior, the detail in which programs of actions are described can impact the degree of variation found. One approach to describing behavioral repertoires and programs of actions is to adopt a multi‐level structure, allowing researchers to pinpoint when and where variation arises. While terminology for these multi‐level structures changes across the literature, we define three levels: *Functional Behavioral Categories, Behaviors* and *Behavioral Elements* (Table 1). Each Functional Behavioral Category can be achieved through a set of Behaviors (Table 1). For example, the Functional Behavioral Category “ Acquire Tool”, could be performed by either collecting an existing tool or by creating a new one. These Behaviors are further specified by combinations of Behavioral Elements (Table 1). The Behavior “create tool” could be completed in various ways, such as tearing parts off the tool (“*stripping*”) or cleanly breaking parts of the material away (“*clipping*”). Choices for behaviors (and their behavioral elements) may not be independent of one another. For example, choice of a tool type may require the individual to select particular grip(s) (e.g., a power grip, as used to hold a hammer, or a precision grip, as with a pen), specific action(s) (e.g., clipping, tearing, picking, or pulling), and the use of different body part(s) (e.g., hands, lips, or teeth).

**Table 1:** Summary of the terminology used to describe the behavioral repertoire of algae fishing by Moyen–Bafing chimpanzees, in increasing levels of granularity.

Term	Definition
Functional Behavioral Category	A broad, higher‐order phase within a sequence of a complete behavior, encompassing essential sequential components of the overall task. Examples include “acquiring a tool”, performing the act of algae “fishing”, and “retrieving algae” from the tool.
	Follows definitions with the same terminology of Byrne et al. 2001; maps to the category level “Activity” in Sanz and Morgan 2009, and “Sequence” in McGrew 1974.
Behavior	A Behavior is a specific action employed by an individual to complete a given Functional Behavioral Category. For example, to fulfill the task of “acquire tool”, an individual may either collect an existing tool or manufacture a new tool.
	Follows definitions with the same terminology of Byrne et al. 2001 and Sanz and Morgan 2009.
Behavioral Element	A Behavioral Element describes the fine‐scale method by which a Behavior is accomplished. For example, the Behavior “tool creation” may involve specific Behavioral Elements such as “clipping” or “stripping” material from vegetation.
	Follows definitions with the same terminology by Byrne et al. 2001; Sanz and Morgan 2009, and Estienne et al. 2017.

*Note:* Functional Behavioral Categories can include multiple Behaviors. Behaviors can be completed using multiple Behavioral Elements.

When comparing tool use within and across species, the behavioral Elements for grip and action have drawn particular interest, as few tool use tasks can be completed without holding and moving the tool. The ubiquity of grip and action in tool use behaviors makes them an important source of comparative data across individuals, tool use tasks, and species. Some grips are functionally optimized for particular tasks: e.g., a power grip (where the tool is held within a fist with the thumb closed over the tool) is ideal for completing a pounding action as it offers the most force (Sperling et al. 1993). Whereas tasks that require the use of fine motor skills are most effectively achieved with a precision grip (Moore and Dalley 2018).

Alongside descriptions of grip and action, hand preference when performing a task is often used to assess and compare the level of dexterity and/or skill required to perform manual actions across tasks or individuals. The consistent decision to use one hand more frequently than the other, across events and locations, suggests that an individual is sacrificing the opportunity to use either hand flexibly depending on local circumstances (e.g., continuing to use their left hand when the location of their grooming partner, or the location and shape of a tree hole they are extracting water from, would sometimes favor the use of their right hand; Boesch 1991). Increasing specialization (or strength of preference), is taken to indicate that a task is more difficult to perform (Boesch 1991). For example, studies of hand preference by wild apes have typically shown strong evidence for individual lateralization in tool use (i.e. consistent right‐ or left‐hand preference) but not in reaching and grooming (Boesch 1991; McGrew and Marchant 1993; Lonsdorf and Hopkins 2005), indicating task‐specialization. Hand preferences are particularly strongly expressed in challenging manual‐tasks that require several years to master (Boesch 1991), such as nut‐cracking (Boesch 1991 Sugiyama et al. 1993), and nettle‐processing (Byrne and Byrne 1991), but in other manual tasks apes retain substantial flexibility (Marchant and McGrew 1998; McGrew and Marchant 2001; e.g., in gesturing: Hopkins and Leavens 1998; Hobaiter and Byrne 2012; although cf., object‐mediated interactions: Hobaiter and Byrne 2012; Forrester et al. 2011).

### 1.1 Exploring Behavioral Units and Programs in Chimpanzee Algae Fishing

Chimpanzees are highly prolific tool users who demonstrate substantial behavioral variation in the expression of a specific tool‐task (e.g., when gathering honey: Sanz and Morgan 2009; Estienne et al. 2019; or when retrieving ants: Humle and Matsuzawa 2002). While the order of Functional Behavioral Categories tends to remain relatively stable within a given tool‐use context (e.g., Estienne et al. 2019), the specific Behaviors and Behavioral Elements employed can vary (Estienne et al. 2019; Humle and Matsuzawa 2002; Boesch et al. 2020), with systematic differences observed both within and between communities (Boesch et al. 2020).

Algae fishing is a recently documented chimpanzee tool use behavior (Boesch et al. 2017), that offers substantial potential for within‐task behavioral variation. Indications of the cognitive challenge algae fishing may pose are evident in the initial description of algae fishing, where Boesch and colleagues (2017) report consistency of hand use within a fishing event and preliminary evidence suggesting population right‐handedness. Although a relatively rare food source in chimpanzee diets, algae consumption has been reported in a handful of populations as a food item that is collected directly using the hands (Sakamaki 1998; Devos et al. 2002; Bueno de Mesquita et al. 2021), or using short (~0.5 m) plant tools to skim algae from the surface of the water (termed “algae *scooping”*: Matsuzawa et al. 1996; Matsuzawa, 2019; Devos et al. 2002). In contrast, algae *fishing* involves the use of variably sized stick tools up to 4 m in length, to retrieve sub‐surface algae growing from the substrate in, at times, deep water (Boesch et al. 2017). While algae scooping tools have been described in detail (Humle et al. 2011), the tool use behavior itself is described more generally and, beyond growing from the surface (in scooping) or substrate (in fishing), there is no further information on the types of algae fished for. We focus on a detailed description of the behavioral repertoire of algae fishing in the same population of chimpanzees studied by Boesch et al. (2017), with the hope that this work facilitates comparison across examples of tool‐based algae consumption and other extractive‐foraging behavior.

Recognizing the importance of a structured systematic approach to behavioral descriptions and the potential for behavioral variation while algae fishing, we build on Boesch and colleagues' (2017) initial report by developing a systematic, detailed behavioral repertoire for algae fishing, including a description of the behavioral steps at three levels of granularity: Functional Behavioral Categories, Behaviors, and Behavioral Elements (following Sanz and Morgan 2009; see Table 1, Figure S1). We then applied this repertoire to define an algae fishing program and its accompanying action‐grammar: the structure and order of behavioral steps when fishing. By describing algae fishing at these structured levels of detail, we offer a framework for behavioral description and facilitate comparison between algae fishing and other tool use tasks across populations and species. We show how both the size of the repertoire and our perceived flexibility of its use can depend upon the levels of descriptive detail applied. We do so in the structure of the behavioral program, and in the selection and combination of Behavioral Elements while preparing to fish, while fishing, and while retrieving algae.

## 2 Methods

### 2.1 Ethics Statement

Ethical approval to collect the data used within this study was granted by the Animal Ethics and Welfare Committee of the University of St Andrews (reference: PS15842; and PS15893). Research was carried out with the permission of the Guinean Ministère de l'Enseignement Supérieur de la Recherche Scientifique et de l'Innovation and the Office Guinéen des Parcs Nationaux et Réserves de Faune. All research was conducted following the legal requirements of the Republic of Guinea and the United Kingdom. All research adhered to the American Society of Primatologists Principles for the Ethical Treatment of Non‐Human Primates.

### 2.2 Study Site and Subjects

Data were collected at the Moyen‐Bafing Chimpanzee Project (MBCP). Located in the former Bakoun Classified Forest of the Moyen‐Bafing National Park in Guinea (11.7267° N −12.7558° W), the MBCP research area (~220 km^2^) encompasses a savanna‐mosaic ecosystem and contains an estimated minimum of ~227 western chimpanzees (*Pan troglodytes verus*; Debetencourt et al. 2024). Algae fishing mainly occurs during October to July and since February 2022, has been opportunistically recorded by the MBCP using camera traps (Bushnell DS‐4K No Glow) and indirect surveys.

### 2.3 Video Coding

Algae fishing occurs at pools of water that are freely accessible in the landscape (i.e. no digging or modification of the environment is required to access the water). We define a “pool” as a body of water often demarcated by the natural features of winding waterways, such as a combination of large boulders, natural ridges, changes in channel depth, vegetated banks, or accumulation of leaf litter. All pools included in these analyses were separated by a minimum of 5 m distance. As many tool use behaviors develop over ontogeny with learning extending into adolescence, we limited the dataset for analysis to adults (15+ years; Goodall 1986), which biases our description to be more conservative as it is less likely to include non‐functional elements. Adults would be expected to be proficient tool users who are more likely to show minimal variation as a result of early trial‐and‐error learning. When describing the algae fishing ethogram, we do so at three nested levels of temporal specificity: a “*Session”*, which is made up of one or more “*Sequences”*, which is, in turn, made up of one or more “*Dips”* (see Table 2). A Session captures the entirety of that instance of tool behavior, a Sequence captures the process to each successful outcome (e.g., eating), and a Dip captures each unique action (or attempt) at retrieving the item. These levels can be generalized to other tool use behavior.

**Table 2:** Summary of terminology used to describe algae fishing by Moyen–Bafing chimpanzees at different levels of temporal specificity.

Term	Definition
Session	A *Session* is marked from when a chimpanzee starts fishing to when they end the behavior. A session starts when a chimpanzee's hand or tool touches the water. A session ends when a chimpanzee: (a) stops fishing, tool making, or eating and leaves the field of view, or (b) engages in another non‐tool related behavior for more than 30 s. A session can involve multiple sequences (*see Sequence)*.
Sequence	A *Sequence* is marked from when a chimpanzee dips their tool or hand into the water and ends when they: (a) eat the algae, or (b) are unsuccessful and decide to terminate their fishing session. A sequence can involve multiple Dips (*see Dip)*.
Dip	A *Dip* is marked from when a chimpanzee's tool enters the water and ends when: (a) the hand leaves the water, or (b) the chimpanzee loses contact with the tool. A Dip can be successful (the chimpanzee retrieves algae) or unsuccessful (the chimpanzee does not retrieve the tool or (sufficient) algae).

As the MBCP video library is large (> 7300 1‐min chimpanzee videos, February 2022 to March 2023), behavioral coding for all examples of algae fishing was not feasible. Therefore, we subsampled the MBCP video library available at the onset of video coding (March 2023), which comprised two algae fishing seasons (recordings form February 2022 to May 2022 and November 2022 to March 2023. When selecting videos for coding, we endeavored to balance the dataset across individuals and locations. Video data were organized by watercourse (i.e. the larger body of running water along which pools were located) and, within each watercourse, by camera trap location (the position of the camera trap), and date of recording. Each camera trap location‐date combination was assigned a unique number, and a subset of video data were randomly selected for subsequent behavioral coding using a random number generator. Within each location‐date dataset, all videos were searched in chronological order for algae fishing. When a video contained algae fishing in which at least one complete Dip by an adult could be coded, we checked the identity of the individual fishing and the number of Dips already coded for that individual. Dips within these videos were then coded unless the individual's contribution to the dataset would exceed 25% of the total number of Dips coded for that watercourse (at the time of coding), in which case that individual was skipped. (In practice, this only occurred for one individual.) Our final coded dataset included 30 camera trap locations distributed across 21 pools, including 62 adult chimpanzees.

For an individual fishing, we coded: date, time, location, session length, individual identity, sex, tool acquisition, tool properties, number of tools used, and the number of other individuals present. A full protocol for behavioral coding, including all variables and definitions is provided in the SI. Coding of an individual fishing continued across all videos to complete the algae fishing Session (Table 2). We considered a new Session to start if there was a pause of 30‐seconds or longer between any of the coded behavioral elements for algae fishing at the same location. Time‐based thresholds when defining a behavior offer a replicable means to describe data, but can also be subjective where there are insufficient data to (as yet) justify their duration through, for example, natural break analysis (Bivand 2022). In this case, we only needed to apply a time‐based threshold to < 1% (1 of 181 Sessions) of the dataset, and so even if 30‐seconds is not reflective of differentiated breaks between “true” sessions, the application of this cut off here is unlikely to have introduced substantial bias as it so rarely occurred. When a video contained multiple adult individuals fishing, all eligible (i.e. adult) individuals were coded. Alongside the capping of individuals, data coding was also capped so that data from a single algae fishing season did not represent more than 75% of the total dataset for that watercourse.

We consider potential sources of bias in our sample using the STRANGE framework (Webster and Rutz 2020), with a detailed description available in the SI. In particular, we describe the possible impacts of using camera trap video data, of an individual's experience on behavioral expression, and of competition in territorial boundary areas on potential inferences drawn about this behavior.

### 2.4 Establishing a Behavioral Ethogram for Algae Fishing

Like all great apes, chimpanzees also show substantial fine motor control relative to that of other primates (Hopkins and Pilcher 2001; Barton and Venditti 2014; Verendeev et al. 2016), with widespread evidence for a strong selective pressure on apes' range of movement and precision in hands and forelimbs (Temerin 1983; Isler, 2005; Hunt 2016; Verendeev et al. 2016; Fannin et al. 2023). Chimpanzees show a similar range of manual motor‐control to humans and employ a similar range of tool‐grips (although their notably shorter thumbs mean that pinch grips are more often expressed between two fingers than finger and thumb (Marzke 1997; Almécija et al. 2015)), with grip choice and proficiency developing with age (Estienne et al. 2019; Malherbe et al. 2024).

We defined the algae fishing ethogram by building on existing extractive foraging frameworks and definitions in apes (Byrne et al. 2001; Boesch and Boesch 1990; Boesch et al. 2009; Sanz and Morgan 2009; McGrew 1974). For variants in behavior that did not conform to existing descriptions and were observed on more than one occasion, we created new definitions and added these definitions to the ethogram. In general, we adopted a bottom‐up approach: coding in as much detail as possible, allowing us to subsequently define categories at different levels of granularity. For example, we discriminated Swivel‐inwards, Swivel‐outwards, and Swivel‐unclear (where the Action was clearly a Swivel but the direction of motion under the water could not be reliably discriminated). We could have assigned Swivel‐unclear Actions to a larger “Unknown” Action category; however, by specifying the information available, this approach to coding allows the observer to retain the option of “lumping” up data to a higher‐order category (e.g., here: Swivel) for potential analysis, minimizing potential data losses.

We assessed the completeness of our description of the behavioral repertoires for two widely described Behaviors (Grip and Action) and their combination into Techniques through asymptote curves. The number of elements in repertoires for each of the Behaviors (Grip, Action) and their combination (Technique) were plotted against the number of observations (Dips) to visualize when in the coding effort new elements were first described.

### 2.5 Dataset Used to Establish Behavioral Ethogram

In total, we coded 1068 min of algae fishing from 608 videos (note that some videos included multiple individuals fishing), which comprised *n* = 181 Sessions, *n* = 2140 Sequences, and *n* = 2431 Dips (see Table 2), from 36 adult males and 26 adult females across the 21 pools (for details of data distribution see Table S1). Within the dataset, each individual contributed a mean of 2.89 Sessions (± 3.81 [SD], range: 1–27) and each pool had a mean of 8.48 Sessions recorded at it (± 8.33 [SD], range: 1–39). We do not include or discuss the potential impact of community identity on behavior as we cannot reliably assign all individuals in our dataset to specific communities, especially since some pools occur in areas used by more than one community (Debetencourt et al. 2024).

To test observer reliability of the coding completed by CW, CH re‐coded a subset of dips (*n* = 144 from *n* = 34 Sessions), representing ~6% of the dataset. We calculated both percentage agreement between raters and Cohen's Kappa (K) on 12 variables (Table S2; a weighted value was used for ordinal variables). Agreement was only marked where both coders indicated the same classification for a variable (e.g. Grip = Power). Data points in which classification of a variable was marked as “Unknown” (e.g., Grip = Unknown) by one rater, were treated as a case of disagreement. Agreement on all variables was above 0.81 K, which is classified as “almost perfect” (Landis and Koch 1977), with all percentage agreements between 88% and 99%.

#### 2.5.1 Hand Preferences

Hand preferences have been described across a wide variety of manual tasks (Boesch 1991; Marchant and McGrew 1998; McGrew and Marchant 2001; Hopkins and Leavens 1998; Hobaiter and Byrne 2012; Forrester et al. 2011) and have been used as a means to compare potential task difficulty (via measures of preference strength, e.g., Boesch 1991). To measure the direction of hand preference while algae fishing, we subset the data to individuals with five or more Sessions with Dips of observable hand‐use and used a hand preference index (HI), calculated as (R‐L)/N. The index varies between −1 and 1, with −1 indicating complete left‐hand use, and 1 indicating complete right‐hand use. Note that no individual was observed to use both hands to perform a fishing Action within a single Dip. We calculated an HI score for per Session per individual. We used these scores to i) calculate strength within a Session for each individual, and ii) to calculate a mean HI score per individual across their fishing Sessions. To measure the strength of hand preference, independently of direction, we calculated an Absolute Hand Preference index (ABS HI), as ABS HI = √(HI^2^), which varies from 0 (no preference) to 1 (complete hand preference in either direction). We calculated an individual's ABS HI across Sessions.

We also calculated an individual's ABS HI score for three of the five Actions (Dunk, Swivel‐inwards, Swivel‐outwards) where an individual had at least 5 Dips with observable body part for that Action (*n* = 1444, *n* = 36 individuals; two actions Sway and Flick were excluded as there were too few individuals with *n* = 5 Dips for each of these Actions, *n* = 1 and *n* = 0 individuals, respectively), and then used these to describe the distribution of ABS HI scores for individual Actions, providing a measure of the flexibility in hand use within each Action. For a full description of the Actions see Results).

## 3 Results

### 3.1 The Behavioral Repertoire and Action‐Grammar of Algae Fishing

We coded 2431 algae fishing Dips, from 62 adult individuals, across 21 pools. Adult fishing Sessions contained a range of 1–119 Sequences (x̄ = 12 ± 18 [SD] Sequences) and lasted between 7 and 3427 s (x̄ = 357 ± 487 s). Most Dips were successful, with algae retrieved in 77% of Dips (*n* = 1866 of 2431 Dips). Moyen‐Bafing chimpanzees employed a diverse repertoire of behavioral units when algae fishing, including: eight Functional Behavioral Categories, and 21 Behaviors produced using 64 Behavioral Elements (see Tables 3 and 4).

**Table 3:** Behaviors used by Moyen–Bafing chimpanzees when algae fishing to complete each functional behavioral category.

Behavior	Required for functional behavioral category?	Description
**Functional behavioral category: acquire tool**		
*Collect tool*	Optional	Chimpanzee enters camera view with a tool or re‐uses an existing tool.
*Create tool*	Optional	Chimpanzee constructs a tool, either by clipping or stripping the tool.
*Adjust tool*	Optional	Chimpanzee adjusts an existing tool, either by clipping or stripping the tool.
**Functional behavioral category (optional): find algae**		
*Inspect water*	Optional	Chimpanzee visually inspects the water before or during the Session.
*Search edge*	Optional	Chimpanzee walks along the edge of the pool while looking towards the water. Must include movement of the chimpanzee along at least part of the edge of the pool while checking the water.
*Head bop inspection*	Optional	Outside of a Dip, the chimpanzee moves their head back and forth or up and down in a fluid repetitive motion while looking at the water.
**Functional behavioral category: prepare**		
*Position self*	Required	Position in which the chimpanzee fishes, including if the chimpanzee uses items in their environment for support.
*Clear debris*	Optional	Chimpanzee clears debris from the surface of the water, either with a tool or with their hand.
**Functional behavioral category: fish**		
*Grip choice*	Required	How the chimpanzee holds the tool.
*Grip where on tool*	Required	Where the chimpanzee holds the tool.
*Action*	Required	Movement of the tool in the water.
*Head bop*	Optional	Within a Dip, chimpanzee moves their head back and forth or up and down in a fluid repetitive motion while looking at the water.
*Sympathetic mouth movements*	Optional	Chimpanzee mouth and lip movements that accompany fine manual motor movements, and that do not appear to have a functional component (e.g., for breathing, eating, vocalizing; *sensu*: Waters and Fouts 2002).
**Functional behavioral category: retrieval**		
*Retrieval action*	Required	Chimpanzee moves algae from end of tool towards mouth.
*Body parts involved in retrieval*	Required	Body parts used by chimpanzee to retrieve algae from tool.
*Algae transfer*	Optional	Algae is transferred from the fishing chimpanzee to another individual.
**Functional behavioral category: eat algae**		
*Eating action*	Required	Chimpanzee removes algae from the tool into their mouth.
**Functional behavioral category (optional): remove debris**		
*Remove debris*	Optional	Chimpanzee removes undesired material from the tool or their mouth.
**Functional behavioral category: finish with tool**		
*Discard tool*	Optional	Session ends when the chimpanzee abandons the tool.
*Tool transfer*	Optional	Session ends when the tool is transferred to another individual.
		Transfer is a Behavior in which a tool is passed directly from one chimpanzee (the primary user) to another, or when another chimpanzee acquires the tool within one second of the primary user discarding it. See Table 4 for further detail on types of transfer.
*Carry tool*	Optional	Session ends when the chimpanzee carries tool away from the pool, out of camera view.

*Note:* While some functional behavioral categories must be completed to successfully fish (required), others can be added or omitted (optional).

**Table 4:** Behavioral elements used by Moyen–Bafing chimpanzees to algae fish.

Functional behavioral categories and their behavioral elements	
**Functional behavioral category: acquire tool**	
***Behavior***: ***collect tool***	
Arrive with a single tool	Chimpanzee enters camera view carrying a tool.
Arrive with multiple tools	Chimpanzee enters camera view carrying two or more tools.
Re‐use others' tool	The chimpanzee uses a tool that another chimpanzee has previously used. To count as re‐use, the coder must have seen another chimpanzee use that tool that day. Where it was unclear if a tool was the same as one used earlier that day, it was marked as a new tool. A chimpanzee can adjust this tool, and it would be marked as re‐use of another individual's tool.
***Behavior***: ***create tool***	
Clip	Use of mouth or hands to remove material in a manner that creates a clean break where the detachment point is relatively smooth and even.
Strip	Use of mouth or hands to create tears using a pulling action, where the detachment point is longer and uneven.
***Behavior***: ***adjust tool***	
Clip	Use of mouth or hands to remove material in a manner that creates a clean break where the detachment point is relatively smooth and even.
Strip	Use of mouth or hands to create tears using a pulling action, where the detachment point is longer and uneven.
**Functional behavioral category: find algae**	
***Behavior: inspect water***	
Inspection on arrival at pool	Before a Session begins, chimpanzee visually inspects the water.
Inspection during Session	During a Session, chimpanzee visually inspects the water.
***Behavior: search edge***	
Search edge on arrival at pool	Before a Session, chimpanzee walks along the edge of the pool while visually inspecting the water.
Search edge during Session	During a Session, chimpanzee walks along the edge of the pool while visually inspecting the water.
***Behavior: head bop inspection***	
Inspection head bop	Outside of a Dip, the chimpanzee moves their head back and forth or up and down in a fluid repetitive motion while looking at the water.
**Functional behavioral category: prepare**	
***Behavior: position self***	
Stand	The chimpanzee has exactly three limbs in contact with the ground (the other will be used when fishing). This includes crouch positions. Configuration while fishing or preparing to fish was always two feet and one hand in contact with ground.
Sit	The chimpanzee's ischial callosities are in contact with the ground, while their upper body is not.
Bipedal	Only the chimpanzee's feet are in contact with the ground.
Lay	Chimpanzee's upper body is in contact with the ground.
Hang branch	The chimpanzee is using one (or more) of their limbs to hang from a plant. Includes any instance where the chimpanzee is using a plant as an anchor. Only observed in combination one of the other Behavioral Elements for this Behavior.
Hang rock	The chimpanzee is using one (or more) of their extremities to hang from a rock. Includes any instance where the chimpanzee is using a rock as an anchor. Only observed in combination one of the other Behavioral Elements for this Behavior.
***Behavior: clear debris***	
Clear debris	Chimpanzee uses a part of their body or the tool to displace debris from the surface of the water.
**Functional behavioral category: fish**	
***Behavior: grip choice*** (see Figure 2 *)*	
Pinch	The tool is held between the sides (not the pads) of two adjacent fingers (typically the index and middle fingers). The tool placement is maintained by pressing the sides of the fingers together. The fingertips do not oppose each other nor contact the tool directly.
Power	The tool is held within the palm, with all fingers curled around it and the thumb closed across the fingers or opposing them, creating a fist‐like configuration. The tool is held in place by the force of squeezing the hand firmly in this fist position.
Precision	The tool is held between two fingers (usually the thumb and index, or thumb and middle fingers). The distal phalanges (fingertips) oppose each other and apply force to hold the tool, a third finger (usually the index or middle) often runs along the tool providing additional support.
Power‐Precision	This grip combines features of the power and precision grips. The tool is held against the palm with the fingers curled around it (as in a power grip), but the thumb (or another finger) is extended along the shaft of the tool rather than folded into the fist. The grip is maintained by squeezing the fingers around the tool, with the extended digit adding support or control.
Finger hold	The tool is held against the palmar surfaces of the fingers, with the fingers curled around the tool, but the thumb and palm does not participate in the grip. The tool is secured by squeezing the curled fingers around the tool, pressing the tool against the bases of the fingers. The thumb does not contact the tool.
***Behavior: grip where on tool***	
1/5 (end)	Tool is held at the part of the tool closest to the chimpanzee.
1/4	Hand is placed at approximately 1/4 (25%) of the total tool length from the end closest to the chimpanzee.
1/3	Hand is placed at approximately 1/3 (33%) of the total tool length from the end closest to the chimpanzee.
1/2	Hand is placed at approximately 1/2 (50%) of the total tool length.
2/3	Hand is placed at approximately 2/3 (66%) of the total tool length from the end closest to the chimpanzee.
3/4	Hand is placed at approximately 3/4 (75%) of the total tool length from the end closest to the chimpanzee. Note this was not observed.
4/5	Hand is placed at approximately 4/5 (80%) of the total tool length from the end closest to the chimpanzee. Note this was not observed.
***Behavior***: ***action*** (see Figure 3 *)*	
Swivel	The distal end of the tool is submerged in water. The chimpanzee then moves the distal tip in a circular motion within the water (i.e. the hand and tool make the motion that would be used to trace a circle or spiral). This movement can be further divided to: *Swivel inwards*: The chimpanzee rotates the tool so that the distal end moves in a circular path towards the animal's body midline. (i.e. left hand + clockwise rotation or right hand + anti‐clockwise rotation). *Swivel outwards:* The chimpanzee rotates the tool so that the distal end moves in a circular path away from the body's midline. (i.e. left hand + anti‐clockwise rotation or right hand + clockwise rotation).
Sway	The distal end of the tool is submerged in water. The chimpanzee moves the tip of the tool that is under the water in a horizontal, side‐to‐side motion (left to right and/or right to left), parallel to the water surface multiple times.
Dunk	The distal end of the tool is inserted vertically downward into the water (along the vertical axis, approximately at a 30°–60° angle to the water's surface), with minimal to no horizontal or lateral movement after being submerged. After reaching a certain depth or briefly remaining submerged, the tool is withdrawn vertically (primarily perpendicular to the surface) from the water.
Flick	The distal end of the tool is inserted into the water and moved in a sharp up‐and‐down motion (i.e. along a vertical axis, perpendicular to the water's surface), so that the distal end moves sharply upward and downward one or more times while submerged. After performing this motion, the tool is withdrawn from the water.
***Behavior***: ***head bop***	
Head bop	The chimpanzee moves their head back and forth or up and down in a fluid repetitive motion while looking at the water.
***Behavior: sympathetic mouth movements***	
Sympathetic mouth movements	Chimpanzee mouth movements that accompany fine manual motor movements, and that do not appear to have a functional component (e.g., for breathing, eating, vocalizing; *sensu*: Waters and Fouts 2002).
**Functional behavioral category: retrieval**	
***Behavior***: ***retrieval action***	
Straight to self	The chimpanzee brings the end of the tool directly to a body part (typically the hand or mouth) without moving the fishing hand's position on the tool. This Action usually occurs using just the fishing hand, but other body parts can be used.
Shimmy tool	The chimpanzee brings the end of the tool to themselves by feeding the tool through their hand(s) or feet. The fishing hand moves away from the proximal end of the tool and closer to the side of the tool that contains the algae.
***Behavior: body parts involved retrieval***	
Left hand retrieval	Left hand assists in bringing the distal end of the tool with the algae back towards the chimpanzee.
Right hand retrieval	Right hand assists in bringing the distal end of the tool with the algae back towards the chimpanzee.
Left arm retrieval	Left arm assists in bringing the distal end of the tool with the algae back towards the chimpanzee.
Right arm retrieval	Right arm assists in bringing the distal end of the tool with the algae back towards the chimpanzee.
Left foot retrieval	Left foot assists in bringing the distal end of the tool with the algae back towards the chimpanzee.
Right foot retrieval	Right foot assists in bringing the distal end of the tool with the algae back towards the chimpanzee.
Mouth retrieval	Mouth assists in bringing the distal end of the tool with the algae back towards the chimpanzee.
Other body part retrieval	Any other body part was involved bringing the distal end of the tool with the algae back towards the chimpanzee.
***Behavior: algae transfer***	
Algae transfer	At least part of the retrieved algae is transferred to another chimpanzee.
**Functional behavioral category: eat algae**	
***Behavior: eating action***	
Side swipe mouth	The chimpanzee closes their mouth around the tool and pulls the tool sideways through their mouth to collect the algae.
Side swipe hand	The chimpanzee closes their hand around the tool and pulls the tool sideways through their hand to collect the algae.
Mouth pick	The chimpanzee picks part of the algae off the tool using their mouth.
Hand pick	The chimpanzee picks part of the algae off the tool using their hand.
Hand assist	The chimpanzee uses their hand to assist in removing the algae, but the algae is not eaten from the assisting hand. For example, the chimpanzee may rest the algae hanging from the tool on their hand as they perform a side swipe mouth. This Action is always in addition to one of the other Behavioral Elements for this Behavior.
Other	None of the Behavioral Elements reported for this Behavior fit the eating Action used.
Hand	The chimpanzee does not use a tool and retrieves algae from the pool directly with their hand.
**Functional behavioral category: remove**	
***Behavior: remove debris***	
Retrieval remove debris	Debris is removed from the tool.
**Functional behavioral category: finish with tool**	
***Behavior***: ***discard tool***	
Release tool on ground	The chimpanzee releases the tool from its grasp so that it falls freely or is placed onto the ground or a rock surface, without handing to another individual, or continued manipulation of the tool after release.
Release tool in water	Similar to “Release tool on ground”, however, the chimpanzee releases the tool from its grasp so that it is located entirely in the water.
Release tool on pool edge	Similar to “Release tool in water”, however the chimpanzee releases the tool from its grasp so that it sits partially in the water and partially on the ground.
***Behavior: tool transfer***	
Tool transfer: intolerant	Transfer is a Behavior in which a tool is passed directly from one chimpanzee (the primary user) to another, or when another chimpanzee acquires the tool within one second of the primary user discarding it. Tool transfer is considered “intolerant” when a tool passes from one chimpanzee (the primary user) to another, either directly or within one second of the primary user discarding the tool, and the primary user appears reluctant to relinquish it. During an intolerant transfer, the recipient is actively attempting to take possession of the tool, such as by reaching for, grabbing, or pulling at it and the primary user exhibits negative behavior—such as facial expressions associated with negative arousal (e.g., bare‐teeth display), gestures associated with negation (e.g., flings), vocalizations indicating negative arousal (e.g., screams, whimpers), or aggressive or submission behavior (e.g., crouching, hitting) during or immediately after the transfer. Intolerant transfers are distinguished from tolerant ones by the clear demonstration of resistance or negative affect on the part of the primary tool user.
Tool transfer: tolerant	Transfer is when the tool is passed to another chimpanzee directly, or within one second, of the primary user discarding the tool. Here, the other chimpanzee is more active in taking the tool, and the primary user shows no negative behaviors once the tool has been transferred.
Tool transfer: Active	Transfer is when the tool is passed to another chimpanzee directly, or within one second, of the primary user discarding the tool. Here, the primary user is more active in giving the tool and also shows no negative behaviors once the tool has been transferred.
Tool transfer: unclear	Transfer is when the tool is passed to another chimpanzee directly, or within one second, of the primary user discarding the tool. Here, it is not clear if the transfer was intolerant, tolerant, or active.
**Behavior: carry tool**	
Carry tool away	The chimpanzee takes the tool and moves with it so that both the tool and the individual carrying it leave the camera's field of view.

#### 3.1.1 Level 1: Functional Behavioral Categories

**Figure 1:**
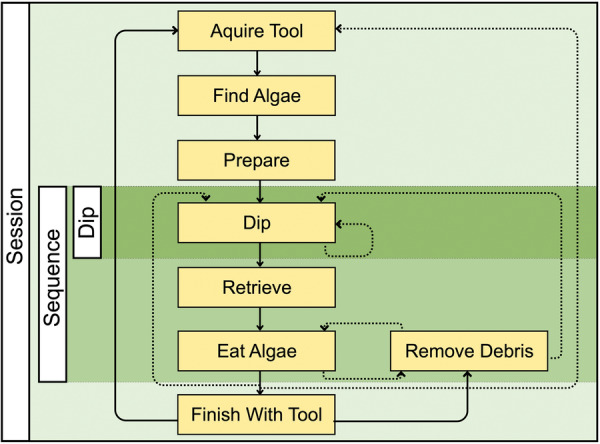
The order of Functional Behavioral Categories completed as part of algae fishing Sessions at Moyen–Bafing. Straight arrows represent the temporal flow of Functional Behavioral Categories. Curved arrows represent repetitions, which could include adjustment of the fishing technique. Green shading indicates the Functional Behavioral Categories that comprise each Session, Sequence, and Dip.

Algae fishing Sessions contained up to eight Functional Behavioral Categories, with “Find Algae” and “Remove Debris” as optional steps. The order of the Functional Behavioral Categories was fixed by the functional requirements of the behavior (e.g., it is impossible to eat algae before first retrieving it). However, chimpanzees demonstrated flexibility in their algae fishing program in two ways: in the presence or absence of steps, and in iterations (repetitions) of steps (Figure 1). Dips were repeated within a Sequence, but rarely (*n* = 182 of 2140 Sequences, < 1%); whereas Sequences were repeated frequently (*n* = 163 of 181 Sessions, 90%), and up to 119 times within a Session. The modal Functional Behavioral Category action‐pattern within a Session was: acquire tool—prepare to fish—complete 11 Sequences (each with a single Dip)—discard tool (Figure 1).

#### 3.1.2 Level 2: Behaviors

A repertoire of 21 Behaviors was available to complete a Session (Table 3). While the inclusion and ordering of the Functional Behavioral Categories was relatively fixed during algae fishing, the inclusion and ordering of Behaviors (collect tool, create tool, adjust tool) within them were more flexible. Some Behaviors (*n* = 7, Table 3) were performed in every case of successful algae retrieval, and are classed as “required” (e.g., Grip and Action, as it is impossible to fish for algae with a tool without holding and moving it), while other Behaviors (*n* = 14, Table 3) were “optional” in the sense that they were not consistently performed (e.g., Inspect Water, or Clear Debris).

#### 3.1.3 Level 3: Behavioral Elements

The size of the repertoire used during algae fishing increased substantially when considering the Behavioral Elements from which an individual could choose to complete a Behavior. Each Behavior was produced by using a choice of up to eight Behavioral Element(s) (range: 1–8; x̄ = 3 ± 2 SD).

All required Behaviors included multiple Behavioral Elements (e.g.,: Grip, see Table 4). The 14 optional Behaviors were either produced using a single Behavioral Element (*n* = 6 of 14), or by choosing from among multiple Behavioral Elements (*n* = 8 of 14; e.g., adjust tool could be completed with either “strip” or “clip”).

### 3.2 Grips

**Figure 2:**
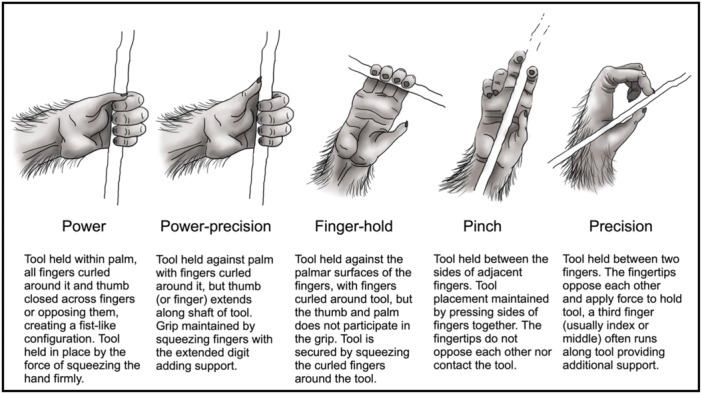
The number of elements of Grip and Action reaching an asymptote over the number of dips coded in our dataset. Our dataset comprises 1652 Dips with observable Actions and 1881 Dips with observable Grips. The asymptote was reached (i.e. reached full diversity) at 306 Dips for Grips, and 102 Dips for Actions.

**Figure 3:**
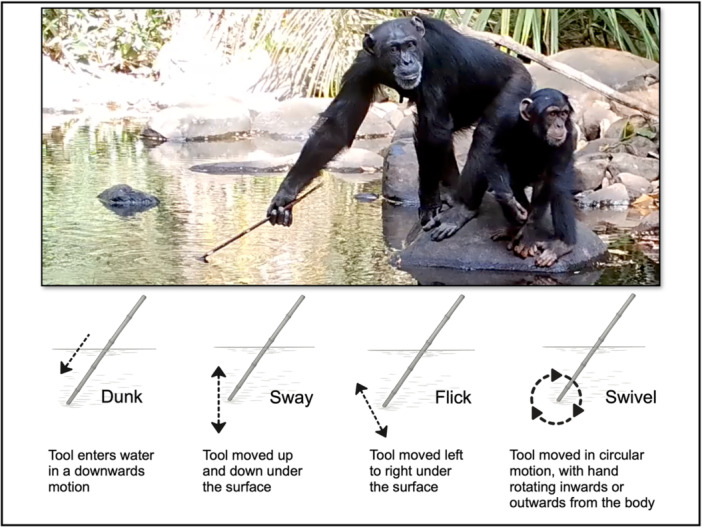
Examples of the five Behavioral Elements for Grip used by Moyen‐–Bafing chimpanzees when holding their tools for algae fishing. See Table 4 for detailed definitions. Illustrations by © Stefano Lucchesi.

We provide a detailed description of the Grips and Actions used in algae fishing, to both understand how algae fishing is executed and to facilitate comparisons with other tool use contexts. The Behavioral Elements described for Grip reached asymptote after only 16% of data coded; *n* = 306 Dips), suggesting that our analysis fully captured the behavioral diversity expressed by the Moyen‐Bafing chimpanzees (Figure 2). Moyen‐Bafing chimpanzees employed five distinct Grips when fishing for algae (Figure 3 and Table 4). Within a Session, individuals used up to four Grip types, although the most commonly observed (i.e. mode) number of Grip types per Session was one.

Adult individuals used predominantly three Grips (mode = 3; across Sessions). Of the 47 adult chimpanzees with more than five Dips recorded with observable Grips, two individuals used all five observed Grips, six used four Grips, 17 used three Grips, 22 used two Grips, and 11 were observed to use one Grip. However, the number of Grips recorded for an individual correlated with the quantity of data recorded for that individual (Pearsons's correlation test: t = 2.1, df = 45, *p* = 0.04), and all individuals observed using only one Grip had fewer than 50 Dips coded in our dataset, suggesting that data density influenced the likelihood of a Grip Behavioral Element being recorded in an individual's repertoire.

### 3.3 Actions

**Figure 4:**
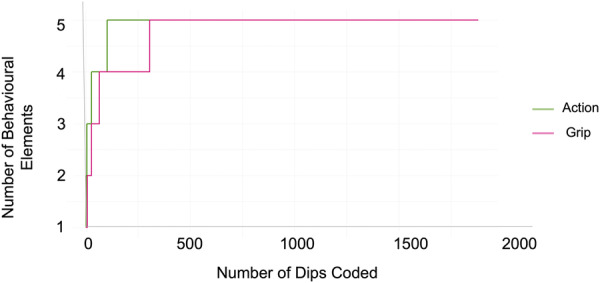
Actions used by Moyen–Bafing chimpanzees while fishing for algae. Note that Swivel was divided into Swivel‐inwards and Swivel‐outwards (and Swivel‐unclear). See Table 4 for detailed definitions. Image credit © Moyen Bafing Chimpanzee Project.

The Behavioral Elements described for Action reached asymptote after ~6% of data coded, suggesting full behavioral diversity was captured (Figure 2). Moyen‐Bafing chimpanzees used four distinct Actions when fishing for algae (Figure 4). Within a Session, chimpanzees were found to use up to four Actions, but the modal number of Actions used was one.

Adult individuals used a modal two Actions (across Sessions). Of the 46 adult chimpanzees with more than five Dips recorded with observable Actions, six individuals used all available Actions, 12 used three Actions, 20 used two Actions and eight chimpanzees were observed to use one Action. However, as for Grips, the number of Behavioral Elements for Action recorded for an individual correlated with the quantity of data recorded for that individual (Pearsons's correlation test: t = 2.4, df = 44, *p* = 0.02), and all individuals observed using only one Action had fewer than 50 Dips coded, suggesting that data density also influenced the likelihood of an Action Behavioral Element being recorded in an individual's repertoire.

### 3.4 Flexibility in the Selection of Behavioral Elements

In our dataset, chimpanzees used 1,468 unique sequences of Behavioral Elements. Successful dips (for which at least 50% of the dip's information could be reliably coded; without unknowns) contained an average of 11 unique Behavioral Elements (±2 SD, range 4‐15). Individuals (with more than 5 dips recorded) used an average of 25 unique Behavioral Elements (± 8 SD; range: 9−44; theoretical maximum; 64). However, the number of Behavioral Elements recorded for an individual correlated with the quantity of data recorded for that individual (Pearsons's correlation test: t = 6.1, df = 40, *p* < 0.01).

### 3.5 Flexibility While Preparing to Fish

In addition to the execution of multi‐step programs of actions, two Functional Behavioral Categories provided indications of behavior consistent with preparedness at the level of the Session. Chimpanzees arrived in view of the camera at the algae fishing location with a tool already in hand (Arrive with tool) on 66% of Sessions (*n* = 99 of 151) where tool acquisition could be coded (*n* = 97 Sessions with one tool; *n* = 2 Sessions with multiple tools). Chimpanzees also left the field of view with their tool in hand (Carry tool away) when finishing their Session in 43% of Sessions (*n* = 66 of 152 sessions where the destination of the tool at the end of a Session could be observed).

Chimpanzees fished from a variety of positions, but most commonly stood with three limbs on the ground (*n* = 1231 Sequences). Chimpanzees occasionally used plants or rocks to hang from (*n* = 178 Sequences), and fished from a combination of places (e.g., arms in the water and legs on ground: *n* = 220 Sequences). Changing fishing position within a Session (*n* = 82 of 181 Sessions, 33%) was relatively common.

### 3.6 Flexibility While Fishing

When fishing, chimpanzees cleared debris from the surface of the water on 3% of occasions (*n* = 50 of 1995 Sequences where we could see if they cleared debris), and head bopped (Table 4; chimpanzee moves head back and forth, or up and down in a repetitive motion while looking at the water) on 18% of occasions (*n* = 342 of 1891 Sequences where the head was visible).

When Dips within a Sequence were repeated, the Dip could be completed using the same Behavioral Element(s) as in the previous Dip or could offer an opportunity for adjustment. We specifically investigated adjustment in the Behavioral Elements for the combination of two Behavior (Grip and Action) as a Technique. Adjustment of Technique within a Dip (i.e. changing Grip or Action while the tool remained submerged in the water) was very rare (*n* = 26 of 2431 Dips, < 1%). Adjustment of Technique between Dips within a Sequence also occurred very rarely (*n* = 59 of 1207 Sequences where Technique could be coded across the entire Sequence, < 1%). In contrast, adjustment of Technique was frequently observed across Sequences within a fishing Session (*n* = 102 of 157 Sessions for which Technique could be coded across the entire Session; 65%).

#### 3.6.1 Strength and Direction of Hand Preferences While Fishing

**Figure 5:**
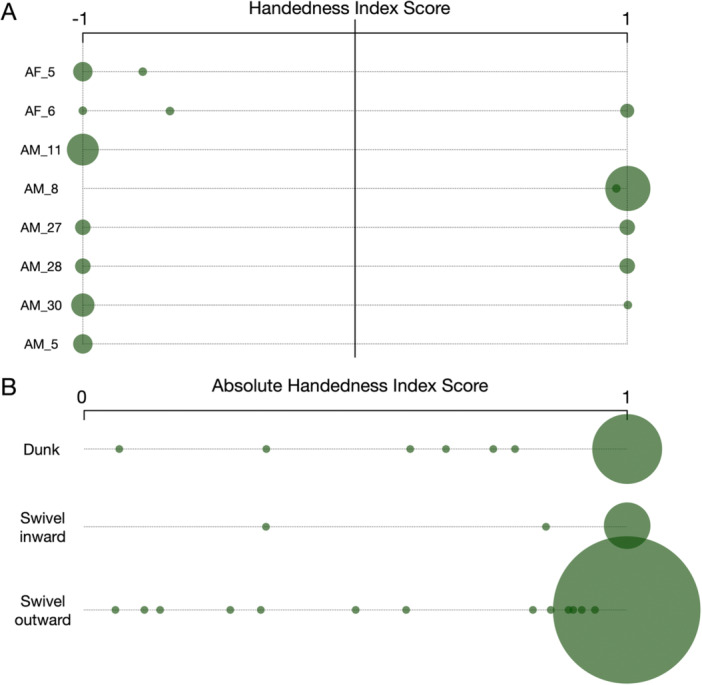
Chimpanzee algae fishing hand preference. (A) Handedness Index for individual chimpanzees′ hand preferences while fishing. A handedness Index (HI) of ‐1 indicates exclusive left‐hand use and 1 indicates exclusive right‐hand use within a Session. We report HI scores for eight adults (two females, six males), each with ≥ 5 Sessions, observed fishing across 15 pools (mean 3.5pools/individual; range = 2‐6, ±1.51 SD). Green circle area scales with the number of Sessions per individual (range = 5–27). For example, Adult Male 8 (AM_8) was observed in 27 Sessions; 26 with a HI score of 1.0 (exclusive right‐hand use), and 1 with an HI score of 0.95 (near‐exclusive right‐hand use). (B) Absolute Handedness Index (ABS HI) scores for fishing Actions. An ABS HI of 0 indicates even use of both hands across Dips for a given Action, and 1 indicates consistent use of the same hand for a given Action. Green circles show ABS HI scores for each Action and are scaled to the number of individuals with that score. Data include 36 adults (16 females, 20 males), each with ≥ 5 Dips per Action (Dunk *n* = 15 individuals; Swivel inward *n* = 8 individuals, Swivel outward *n* = 32 individuals, individuals can contribute to multiple Actions). For example, for Swivel Outwards, 32 individuals met this criterion: 19 had an ABS HI of 1.0 (fully consistent hand use), and 13 had scores from 0.06 to 0.94, indicating near‐even to near‐consistent hand use, respectively.

Individual chimpanzees were strongly lateralized within a session, exhibiting a consistent hand preference (n = 8 individuals; ABS HI: range = 0.67‐1.00, x̄ = 0.99 ± 0.04 SD), but did not show a consistent direction of hand preference across Sessions (HI: range = −1.00‐1.00, x̄ = 0.02 ± 1.00 SD; Figure 5A).

We found minor variation between the Actions in the consistency of an individual's hand preference when performing an Action. Overall, most individuals used the same hand to perform the same Action. However, individuals showed a slightly larger absolute hand preference for the Action Swivel‐inwards (*n* = 2 ABS HI range 0.33–0.85; *n* = 6 ABS HI = 1.0) as compared to the Actions Dunk (n = 6 ABS HI: range 0.07–0.79; *n* = 9 ABS HI = 1.0), and Swivel‐outwards (*n* = 13 ABS HI range 0.06–0.94; *n* = 19 ABS HI = 1.0; Figure 5B). With the mean ABS‐HI values for Swivel‐in (ABS HI x̄ = 0.90 ± 0.23 SD), also slightly higher across individuals than Swivel‐out (ABS HI x̄ = 0.82 ± 0.30 SD), and Dunk (ABS HI x̄ = 0.81 ± 0.29 SD).

### 3.7 Flexibility While Retrieving Algae

Chimpanzees used their hands, feet, mouth, and arms to bring the tool back towards themselves during algae retrieval. It was more common during retrieval Actions for chimpanzees to use multiple body parts than it was to use multiple body parts during fishing (*n* = 360 of 1848 Sequences with observable retrieval Actions; 19.5%; Chi‐square test: χ^2^ = 380.95, df = 1, *p*‐value < 0.01).

When retrieving algae, the most common Action was to bring the end of the tool straight to the mouth (*n* = 1372 of 1856 Sequences with observable retrieval Actions; 73.9%). Chimpanzees used both their hands and their mouths to remove algae from the end of the tool, typically by swiping the tool through their mouth or hand (*n* = 1606 of 1859 Sequences with observable eating Actions; 86.4%) rather than by picking algae off the tool with their mouth or hand (*n* = 253 of 1859 Sequences with observable eating Actions; 13.6%; Chi‐square test: χ^2^ = 1730.9, df = 1, *p*‐value < 0.01). Chimpanzees also occasionally removed debris from algae they retrieved while eating it (*n* = 50 of 1995 Sequences where this could be reliably coded; 2.5%).

## 4 Discussion

Moyen‐Bafing chimpanzees employ a varied repertoire of behavioral units when fishing for algae: eight Functional Behavioral Categories and 21 Behaviors, produced using 64 Behavioral Elements. The order of the program of actions was largely fixed at the highest‐level of categorization (i.e. Functional Behavioral Categories) but, even at this hierarchical level, algae fishing in this population included two optional steps leading to four main variations in the observed program of actions, with further variation generated by the use of iterations (i.e. repetitions of both individual Functional Behavioral Categories and sets of multiple Functional Behavioral Categories). At the level of Behaviors, the number of program variations generated by the combination of units was orders of magnitude larger. As in other tool use behavior, chimpanzees employed a set of different Grips. However, unlike most other tool tasks (Sanz and Morgan 2010; e.g., nut cracking with stone tools: Biro et al. 2006; leaf‐folding for drinking water: Tonooka 2001), adult chimpanzees retained and used a variety of Actions when fishing for algae, suggesting that there is unlikely to be a single “optimal” solution across individuals and occasions in this dynamic problem space.

Comparison across types of tool use—even within chimpanzees, let alone across species—is made challenging where the frameworks used to describe behavioral variation vary in specificity and completeness (Estienne et al. 2017; Grund et al. 2024). Here we provide a framework to facilitate comparison, by incorporating features of tool use description from across studies (Byrne et al. 2001; Boesch and Boesch 1990; Boesch et al. 2009; Sanz and Morgan 2009; McGrew 1974; Estienne et al. 2017) and using these as a basis to systematically describe a new tool behavior at multiple levels of detail. In doing so, we not only characterize a new tool behavior in detail, but we provide a basis from which to start to quantify variation in this behavior (algae fishing) relative to that reported for other chimpanzee tool tasks.

For example, in a study of underground honey extraction, Estienne and colleagues (2017) identify three Phases (exploration, tool manufacture, extraction) and 14‐17 Behaviors (termed Actions) which include a combination of what we term Behavior and Behavioral Elements (Table 5). They estimate that Action repertoire size in other studies of tool use varied from 2 to 13 of their Actions (including leaf sponging, underground and arboreal honey extraction, nut cracking, and elevated and subterranean termite fishing; Estienne et al. 2017; Tonooka 2001; Sanz and Morgan 2010; Sousa et al. 2009; Inoue‐Nakamura and Matsuzawa 1997; Nishida and Hiraiwa 1982, Table 5). In comparison, algae fishing appears to offer substantial potential for behavioral variation, with large repertoires of units at all levels of granularity, and a relatively long program (cf., other tool uses: Estienne et al. 2017; Sanz and Morgan 2010; Lonsdorf 2005; Biro et al. 2006; Boesch et al. 2020, Table 5).

**Table 5:** Comparison of repertoire sizes in different tool tasks used by chimpanzees, adapted from Estienne et al. (2017).

Tool‐task	Higher order category^h^	Detailed category^i^
Algae fishing (this study)	Functional Behavioral Category (7)	Behaviors (21) *Behavioral Elements (64)* j
Underground honey extraction^a^	Transitions (3)	Actions (14‐17)
Arboreal honey extraction^e^, ^h^	Transitions (3)	Actions (18)
Leaf Sponging^e^, ^h^	Transitions (2)	Actions (7)
Leaf Sponging^e^, ^i^	NA	Actions (6)
Leaf Sponging^e^, ^j^	NA	Actions (7)
Nut Crackinga,e	Transitions (3)	Actions (4)
Nut Crackinga,f	NA	Actions (26)^j^
Termite Fishing (elevated nests)a,g	NA	Actions (3)
Termite Fishing (elevated nests)^e^, ^h^	Transitions (2)	Actions (13)
Termite Fishing (subterranean nests)^e^, ^h^	Transitions (3)	Actions (16)

^a^Estienne et al. (2017).

^b^Sanz and Morgan (2010).

^c^Sousa et al. (2009).

^d^Tonooka (2001).

^e^ Boesch and Boesch (1982).

^f^Inoue‐Nakamura and Matsuzawa (1997).

^g^Nishida and Hiraiwa (1982).

h Transitions refers to the number of steps in a sequence of tool‐use behavior; these share overlap with—but are different to—our Functional Behavioral Categories, which can be skipped (minimum to complete the algae fishing sequence = 5), and each Functional Behavioral Category can contain multiple Behaviors, with transitions between them. Thus, the minimum number of transitions in algae fishing would be five.

i In Estienne et al. Actions are similar to our category of Behavior, which in our case includes Actions, Grips, Retrieval Action, etc.

j Note that some of the studies reported in Estienne et al. with a large number of Actions (by their definition), include items that we would classify as Behavioral Elements (of which algae fishing has *n* = 64).

While there is no objectively “correct” level of analysis—these are sensitive to the question being asked (and the data available)—the use of fine‐grained and consistently structured descriptions of behavior allows for easier comparison across studies (as units of analysis can be reconstituted or “translated” across samples; Grund et al. 2024; Mielke et al. 2024). Chimpanzee algae fishing can, at one level of description, have relatively little variation (with flexibility limited to omissions or repetitions) and, simultaneously, thousands of program variants at other levels of description (where dozens of different Behavioral Elements are selected from to complete a Behavior). The systematic inclusion of essential Behaviors (e.g., in tool use: Grip, Action, and their combination as Technique) at the same level of detail (Behavioral Elements) across studies could provide a key foundation in which to situate the relative flexibility of a behavior within and across species (Wessling et al. 2026). Moreover, systematic descriptions are also important for accurate comparisons in applied conservation actions: for example, in understanding population resilience in the face of rapid environmental change, or in defining target populations for conservation activities (Wessling and Surbeck 2022; Wessling et al. 2025a).

Regular within‐individual adjustment of Techniques within an algae fishing Session suggests that chimpanzees can respond dynamically within an algae fishing event (e.g., as the availability of algae in a location diminishes). By contrast, comparable flexibility in Technique was not seen at the level of a Dip or Sequence. Similarly, planning to bring a tool with you (or take it on afterwards), could only be observed at the level of a Session. Other types of behavioral flexibility can be detected at the level of Sequences. For example, chimpanzees regularly incorporated Behaviors such as clearing the surface of the water or head bopping movements, likely in response to the challenges of an aquatic niche such as surface obstructions, or visual challenges of diffraction and sun glitter (Loew and McFarland 1990; Temple et al. 2010).

The problem being solved when fishing for algae changes both across pools and within pools over time. Across Sessions, pool depth, water opacity, water current strength, algae growth depth, algae density, access to the pool, and the social environment, among others, can change. Within a Session, algae growth depth, algae density, access to the pool and the social environment can change. To be able to successfully acquire algae, chimpanzees must navigate a dynamic problem space by selecting and executing the appropriate combination and sequence of Behavioral Elements—choices that appear particularly extensive for algae fishing (Table 4), as compared to those made in other tasks, given their repertoire sizes and program lengths (Estienne et al. 2017; Sanz and Morgan 2010; Lonsdorf 2005; Biro et al. 2006; Boesch et al. 2020; Table 5). If each choice represents a distinct decision step that must be integrated into a single outcome, it would suggest that algae fishing is a cognitively demanding task. While the population repertoire comprises thousands of *program* variants, real‐time decision‐making is shaped by immediate circumstances, and—at any one time—many of these variants may be unavailable (e.g., some spots from which to fish around the pool may be already occupied, or debris may only need cleared on the first, but not subsequent, Sequences). These contextual constraints likely simplify the load on working memory and decision making, thereby moderating the overall cognitive challenge. Although algae fishing has, at present, only been described in Moyen‐Bafing chimpanzees, “algae scooping” is present at other chimpanzee communities (Matsuzawa et al. 1996; Matsuzawa, 2019; Devos et al. 2002). Algae scooping involves collecting algae from the water's surface using small stick tools (Matsuzawa et al. 1996; Matsuzawa, 2019; Devos et al. 2002). While algae fishing and algae scooping address a similar general problem (ie using tools to obtain algae) and may constitute a continuum rather than a strict dichotomy, algae fishing likely involves greater variability due to the wider range of tool lengths and water depths involved. A systematic description of algae scooping within a comparable framework will allow us to investigate if, where, and how these behaviors differ.

For two of the most widely described aspects of tool‐use: Grip and Action, and their combination into 22 algae fishing Techniques, the repertoires of units used in algae fishing quickly reached an asymptote at the population level. That Techniques quickly reached asymptote suggests that, at our most fine‐grained level of description (Behavioral Elements), the full extent of variation in the repertoire was sufficiently/reliably captured. While we described variation between individuals in their repertoire size, these differences appeared very sensitive to sample size, suggesting that further data collection per individual would likely result in more, if not all, the population‐level Behavioral Elements being described in their individual repertoire. As a result, individual variation in the use of Grips, Actions, and Techniques likely reflects flexible, case‐by‐case responses to specific challenges—shaped by the physical and social environment or by personal preferences—rather than an inability to perform particular behaviors. Most examples of chimpanzee tool use appear to have a clear optimal Action on which individuals converge (Sanz and Morgan 2010; e.g., nut cracking with stone tools: Biro et al. 2006; leaf‐folding for drinking water: Tonooka 2001). In contrast, chimpanzees' exploitation of their large repertoire of Actions in algae fishing supports the possibility that chimpanzees may benefit from retaining flexibility in this dynamic problem space.

One means to assess the physical and cognitive challenge of producing manual actions is the extent to which individuals show specialization through stronger hand preferences (rather than retaining the flexibility to use either hand in response to variation in the physical environment; Leavens et al. 2001; Fitch and Braccini 2013; Boesch 1991), with hand specialization suggested to benefit efficiency (McGrew 1999). Our findings support Boesch and colleagues' (2017) report that individual chimpanzees were near‐perfectly lateralized when fishing for algae (i.e. they do not swap hands during a Session). In this respect, individuals show comparatively strong hand preferences when algae fishing, similar to those reported for other cognitively challenging tool‐tasks such as nut cracking with paired stone tools (Bossou: ABS‐HI = 1.0, Humle and Matsuzawa 2009), and as high or higher (ABS‐HI = 0.7–1.0) than those reported for a range of tool‐task behavior (ABS‐HI = 0.5‐0.8; Humle and Matsuzawa 2009).

However, we do not find evidence of population‐level right handedness. In part, this discrepancy could be explained by our focus on adults: a previously reported right‐handed bias was most prominent in immature individuals, with no evidence for a bias in adults (Boesch et al. 2017). We find that across Sessions, only half of the individuals could be described as demonstrating a strong hand preference, and only one of eight was right‐hand biased. Our data meet, at most, Level 2 (of a possible 5) in McGrew and Marchant's (2001) framework for population‐level laterality (with humans at Level 5; McGrew and Marchant 2001). Chimpanzees' choice of left or right hand when fishing for algae may be shaped, in part, by the physical affordances of the local environment. Environmental constraints may limit the ability to maintain a hand preference while using tools, as found for other behavior, such as ant fishing (Marchant and McGrew 2007). We also found some consistency in an individual's hand preference when performing a particular fishing Action, across different Sessions most individuals preferred to use the same hand to produce the same Action, with the greatest consistency in Swivel‐inwards Actions. Together these findings suggest that there may be some benefits from task specialization in algae fishing—but that these operate most strongly at the level of specific Behavioral Elements, and that individuals retain the ability to produce even fine‐grained motor skills with either hand when needed.

Finally, Moyen‐Bafing chimpanzees typically (66%) arrived in view with a tool already in hand. Algae fishing tools can be over 4 m in length (Boesch et al. 2017), and while bamboo and wood may be relatively lightweight materials, longer tools are likely inconvenient to carry. The use of camera trap data means that we cannot assess the distance across which the chimpanzees were transporting their tools, and these cases could reflect relatively minor adjustments in position around a pool or the sourcing of the tool from a nearby plant in view of the pool. Most camera trap models have a trigger distance of under 40 m making this type of data collection not well suited to assessing whether chimpanzees carry their tools across longer distances. However, at other sites chimpanzees have been observed carrying tools over dozens of meters suggesting that they are indeed out‐of‐sight of the problem when gathering tool material (nut‐cracking in Taï Forest: Boesch and Boesch 1984; termite‐fishing in Issa: Almeida‐Warren et al. 2017), and that chimpanzees have a mental representation of the problem they are planning to solve (Boesch and Boesch 1984; Byrne et al. 2013; Almeida‐Warren et al. 2017; Musgrave et al. 2024).

## 5 Conclusion

The relatively large repertoires of Behaviors and Behavioral Elements described of this behavior, alongside their hierarchical organization in a program of actions that shows variation in structure, supports the body of evidence that chimpanzees respond flexibly to cognitively challenging tasks (Sambrook and Whiten 1997; Coolidge and Wynn 2001). Our ability to situate the relative behavioral flexibility shown in a task, or within and between species, remains limited by differences in the ways in which behavior are described, with important implications for our ability to address questions relating to cognitive and behavioral evolution and adaptability. Here, we provide a systematic description of a new tool task in chimpanzees at multiple levels of detail. In doing so we facilitate comparison with other tool tasks and show that adult chimpanzees continue to use a particularly diverse set of Techniques when fishing for algae, suggesting that they benefit from retaining multiple solutions in this dynamic problem space.

## Author Contributions

**Charlotte Wiltshire:** conceptualization, investigation, writing – original draft, methodology, validation, visualization, writing – review and editing, formal analysis, data curation. **Erin G. Wessling:** conceptualization, investigation, funding acquisition, writing – original draft, methodology, validation, visualization, writing – review and editing, formal analysis, project administration, data curation, supervision, resources. **Liran Samuni:** conceptualization, investigation, writing – original draft, funding acquisition, methodology, validation, visualization, writing – review and editing, formal analysis, project administration, data curation, supervision, resources. **Catherine Hobaiter:** conceptualization, investigation, funding acquisition, writing – original draft, methodology, validation, visualization, writing – review and editing, formal analysis, project administration, data curation, supervision, resources.

## Conflicts of Interest

The authors declare no conflicts of interest.

## Supporting information

Supporting File 1

## Data Availability

The data that support the findings of this study are available from the corresponding author upon reasonable request. The data that supports the findings of this study are available upon reasonable request from the corresponding authors. All code for the analyses is available in our Github repository: https://github.com/Wild‐Minds/AlgaeFishing.
