# Supplementary material for: The Forms and Structures of Chimpanzee Algae Fishing
Source: Am J Primatol. 2026 Jun 8;88(6):e70164. doi: 10.1002/ajp.70164 (PMC13244816; doi:10.1002/ajp.70164)
Supplement: Supplementary file 1 — Supporting File 1 [file AJP-88-e70164-s001.docx]

**SUPPLEMENTAL MATERIALS**

A full protocol for behavioral coding, including all variables and definitions is available here: <https://github.com/Wild-Minds/AlgaeFishing>

*STRANGE: assessing potential sample biases.*

The STRANGE framework is a framework to report socio-ecological features which may impact findings from the study sample (Webster & Rutz, 2020). STRANGE works to highlight sampling bias by addressing seven categories which may be a source of bias: Social background, Trapability and self-selection, Rearing history, Acclimation and habituation, Natural changes in responsiveness, Genetic make-up, and Experience. Below, we summarize each category for Moyen-Bafing chimpanzees.

**Social Background:** At present, limited information is available regarding the social background of Moyen-Bafing chimpanzees. The communities of Moyen-Bafing chimpanzees are estimated to be ~37-58 individuals (Debetencourt *et al.* 2024), relatively typical community sizes for the subspecies (Wilson *et al.,* 2014). Preliminary results suggest an adult sex ratio of 1.32 (Debetencourt *et al.,* 2024), which is higher than that described for forest populations (Badihi *et al.,* 2022) but is similar to other savannah populations (Wilson *et al.,* 2014). A larger sex ratio may result in greater male-male competition (Wilson *et al.,* 2014; Pruetz *et al.,* 2017); however, Western chimpanzees are suggested to be more gregarious and tolerant than other chimpanzee subspecies (Boesch *et al.,* 2008; Wilson *et al.,* 2014). As a result, Moyen-Bafing chimpanzees may not experience increased competition and aggression as a result of sex-based demographics, as compared to other studied chimpanzee communities.

**Trapability and self-selection:** This study uses video data collected using camera traps. Chimpanzees who have had very little exposure to humans and/or human artefacts may react with caution to new objects in their environment. At other chimpanzee field sites, individuals have shown variation in their response to camera traps, which may result in some individuals being less likely to be recorded (Caravaggi *et al.,* 2020). Chimpanzees at the MBCP have been exposed to camera traps since 2013 as part of the Pan-African Program (Debetencourt *et al.,* 2024). They show interest in the cameras and continue to acknowledge their presence when passing by. We recognize that camera trap data may lead to some level of self-selection, but likely less so than direct observation (Hobaiter *et al.,* 2017) and we have recorded a large number of individuals on the camera traps, including all age and both sex classes.

Alongside self-selection, our choice in position of the camera traps may impact the individuals included in our study. When choosing camera location, researchers have large areas in which to search for evidence of algae fishing. When and where algae will grow remains difficult to predict, making it possible that some pools were not camera trapped when chimpanzees were fishing.

**Rearing History:** All chimpanzees are wild born, with no direct influence from humans.

**Acclimation and habituation:** None of the individuals included in this study were habituated to direct observation.

**Natural changes in responsiveness:** As wild animals, individuals had complete freedom when deciding when and how to fish for algae. While behavioral changes may occur when algae fishing over time, these do not negatively impact our exploration of variation when algae fishing. Individuals fishing in pools located at boundary areas between community territories may express higher levels of arousal because of the risk of agonistic (or even lethally aggressive) behavior from neighboring groups (Wilson *et al.,* 2003; Mitani & Watts, 2005). As territories for the communities have not yet been fully described, it remains unclear which specific pools were located in boundary areas and/or were accessed by multiple communities. Increased arousal may lead to variation in algae fishing expression; however, we focused our description of the repertoire and program of actions across individuals at the population level.

**Genetic Makeup:** Female chimpanzees emigrate to a new community during adolescence, and there are no site-specific barriers to transfer of females between communities at MBCP, making the influence of systematic genetic differences on algae fishing behavior less likely.

**Experience:** Chimpanzees at Moyen-Bafing have had camera traps present in their territories since 2013 (Debetencourt *et al.,* 2024) and therefore have long-term experience with camera traps. This exposure reduces the impact of self-selection or unusual behavior. As experience in algae fishing may vary across areas and individuals, we limited our data to adult individuals, and included 62 individuals fishing from pools across a diverse set of locations in the study area.

**Table S1: Dataset of adult chimpanzee algae fishing by pool.** The number of adult individuals in the dataset observed fishing in each pool, along with how many Dips were coded for that pool. Total number of adult individuals in the dataset is 62, but the same individual can fish in more than one area.

| Pool | No. Males | No. Females | No. Dips |
| --- | --- | --- | --- |
| 1 | 5 | 2 | 487 |
| 2 | 1 | 3 | 9 |
| 3 | 2 | 1 | 10 |
| 4 | 3 | 0 | 7 |
| 5 | 3 | 4 | 497 |
| 6 | 1 | 0 | 12 |
| 7 | 3 | 1 | 233 |
| 8 | 5 | 3 | 228 |
| 9 | 5 | 2 | 254 |
| 10 | 5 | 3 | 85 |
| 11 | 4 | 1 | 171 |
| 12 | 3 | 4 | 50 |
| 13 | 1 | 0 | 6 |
| 14 | 2 | 0 | 16 |
| 15 | 1 | 3 | 19 |
| 16 | 2 | 1 | 15 |
| 17 | 6 | 2 | 70 |
| 18 | 4 | 1 | 57 |
| 19 | 2 | 2 | 14 |
| 20 | 4 | 1 | 17 |
| 21 | 2 | 4 | 158 |

**Table S2.** Interobserver reliability values across 12 variables. We included 144 Dips within 38 Sessions, where n= number of times that Variable was coded for Interobserver reliability.

| **Variable** | **n** | **Cohen’s Kappa (SE)** | **95% confidence** | **Percentage agreement** |
| --- | --- | --- | --- | --- |
| Arrive tool^1^ | 35 | 0.930 (0.046) | 0.862 – 1.000 | 97.1 |
| Tool create^2^ | 37 | 0.881 (0.065) | 0.754 – 1.000 | 91.9 |
| Body part fish | 144 | 0.927 (0.032) | 0.864 – 1.000 | 96.5 |
| Grip | 144 | 0.847 (0.034) | 0.781 – 0.914 | 88.2 |
| Body position | 144 | 0.975 (0.018) | 0.940 – 1.000 | 98.2 |
| Action | 144 | 0.923 (0.028) | 0.868 – 0.978 | 95.1 |
| Head bop | 144 | 0.813 (0.055) | 0.706 – 0.920 | 92.4 |
| Body part retrieve | 144 | 0.961 (0.019) | 0.923 – 0.999 | 97.1 |
| Retrieval | 144 | 0.937 (0.028) | 0.882 – 0.991 | 96.5 |
| Eating technique | 144 | 0.912 (0.035) | 0.844 – 0.980 | 95.1 |
| Tool use end | 38 | 0.928 (0.049) | 0.882 – 1.000 | 94.7 |
| Clear debris | 144 | 0.971 (0.029) | 0.915 – 1.000 | 99.3 |

^1^ Weighted Kappa as ordinal categories


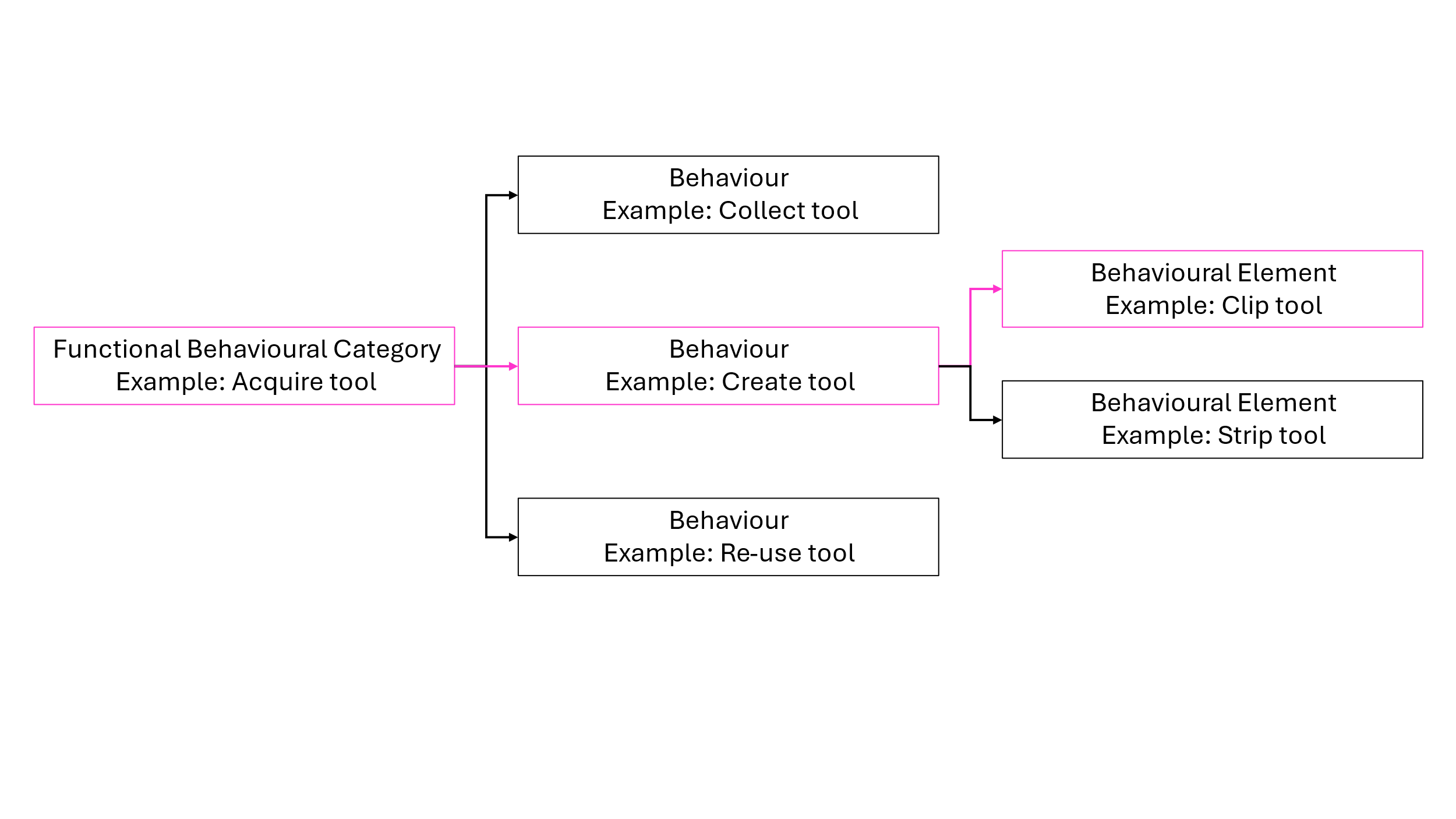
**Figure S1:** Schema of algae fishing program with three levels: Functional Behavioral Categories, Behaviors, and Behavioral Elements
